# Mean Brain Weight among Autopsy Cases at the Department of Forensic Medicine of a Tertiary Care Centre: A Descriptive Cross-sectional Study

**DOI:** 10.31729/jnma.7162

**Published:** 2022-03-31

**Authors:** Jwala Kandel, Dikshanta Pokharel

**Affiliations:** 1 Department of Forensic Medicine, Nobel Medical College Teaching Hospital, Biratnagar, Morang, Nepal; 2 Department of Forensic Medicine, Koshi Hospital, Biratnagar, Morang, Nepal

**Keywords:** *autopsy*, *brain*, *organ weight*

## Abstract

**Introduction:**

Weight of the brain is an important diagnostic criterion during autopsy. Normal variations in brain weight among various population demands for population-specific study. The aim of this study was to find the mean brain weight among autopsy cases at a tertiary care centre.

**Methods:**

A descriptive cross-sectional study was conducted among autopsy cases in the Department of Forensic Medicine of a tertiary care hospital from July 2019 to July 2021. The approval for the study was obtained from the Institutional Review Committee (Reference number: 550/2019). Convenience sampling was done. Brain was dissected following standard autopsy technique and weight was measured by an electronic digital weighing scale (5 kg/0.01 gm). Statistical Package for the Social Sciences version 20.0 was used for data analysis. Point estimate at 95% Confidence Interval and descriptive statistics were used to express results in frequency, mean, standard deviation, range, and percentage.

**Results:**

The mean brain weight for 394 autopsy cases was 1272.38±144.07 grams (1258.15-1286.60 at 95% Confidence Interval). For male and female it was 1322.47±140.22 grams and 1221.27±129.55 grams respectively. Maximum attainment of brain weight was found at 21 to 30 years age group for both sexes. Out of 394 cases, 199 (50.50%) were males and 195 (49.50%) were females. In all the age groups, male brain weight was greater than that of female.

**Conclusions:**

The mean weight of the brain derived from the present study is found to have variable values in comparison to other studies done under similar settings.

## INTRODUCTION

The weight of the brain is regularly used as a criterion to differentiate normal from pathological conditions during post mortem examination. It is an important factor indicating an abnormality in the autopsy as well as clinical settings. Any deviation from normal weight is suggestive of an underlying pathological condition resulting from disease or injuries. Thus, it helps in determining the cause and manner of death which is vital for any medico-legal case.^1,2^

Numerous such studies have been conducted for various populations, which may not be helpful in the Nepalese context as there are genetic, dietary, environmental, and various other influences on organ weight.^3,4^

The main objective of this study was to find the mean brain weight among autopsy cases at a tertiary care centre.

## METHODS

This was a descriptive cross-sectional study conducted in the Department of Forensic Medicine of a tertiary care hospital from July 2019 to July 2021. Cases of autopsies brought in Koshi Zonal Hospital, Nepal were included in the study. Ethical clearance for the study was taken from the Institutional Review Committee of Nobel Medical College (Reference number: 550/2019). The study included brain weights of Nepalese deceased, taken during post-mortem examination. Family members of the deceased were pre-informed regarding the use of autopsy data for study purpose and verbal consent was obtained. The cases of death due to diseases and injury over the brain, decomposed bodies, cases with a history of IV fluid, and blood transfusion were excluded from the study as these factors would cause differences in the normal brain weight.

The sample size was calculated using the formula:

n = (Z^2^ × σ) / e^2^

  = (1.96^2^ × 75.67^2^) / 8^2^

  = 344

Where,

n = minimum required sample size
Z = 1.96 at 95% of Confidence Interval (CI)
σ = standard deviation calculated from minimum and maximum value^5^
e = margin of error

However, 394 cases were included in the study using a convenience sampling method. The brains of the deceased were dissected following standard autopsy techniques.^6^ Dural covering and attached vessels were completely stripped off. Weight of the brain consisting of cerebral hemispheres, cerebellum, and midbrain were measured with the same electronic digital weighing scale (5 kg/0.01 gm) in all the cases.

Data entry and analysis was done by Statistical Package for the Social Sciences version 20.0. Point estimate at 95% Confidence Interval and descriptive statistics were used to express results in frequency, mean, standard deviation, range, and percentage.

## RESULTS

The mean brain weight for 394 autopsy cases was 1272.38±144.07 grams (1258.15-1286.60 at 95% Confidence Interval). Mean brain weights for male and female were 1322.47±140.22 grams and 1221.27±129.55 grams respectively. Out of 394 cases, 199 (50.50%) were males and 195 (49.50%) were females (Table 1).

**Table 1:** Descriptive statistics for male and female brain weights (n= 394).

Sex	Mean±SD (degrees) (Minimum to maximum)	n (%)	Range (grams)
Male	1322.47±140.22 (936-1588)	199 (50.50)	652
Female	1221.27±129.55 (925-1555)	195 (49.50)	630

The maximum mean brain weight of 1371.65±118.67 grams is noted in the 21 to 30 years age group whereas the least 971.53±34.78 grams was seen in the 1 to 10 years age group. Male deceased were between 9 to 92 years while females were between 7 to 93 years. In all the age groups, male brain weight was greater than that of females (Table 2).

**Table 2:** Descriptive statistics of brain weights (grams) for various age groups (n = 394).

Age group (years)	Mean±SD	n (%)	Minimum	Maximum	Range
1-10	971.54±34.78	13 (3.30)	925	1032	107
11-20	1268.15±148.10	53 (13.45)	1003	1487	484
21-30	1371.66±118.67	76 (19.29)	1150	1588	438
31-40	1336.40±105.73	62 (15.74)	1110	1530	420
41-50	1317.53±112.35	55 (13.96)	1105	1502	397
51-60	1249.33±107.09	54 (13.71)	1065	1455	390
61-70	1196.67±97.72	39 (9.90)	1021	1375	354
71-80	1150.45±81.86	31 (7.86)	1015	1302	287
81-90	1121.13±85.46	8 (2.03)	1001	1246	245
>90	1048.00±99.54	3 (0.76)	955	1153	198

## DISCUSSION

The mean brain weight in the present study was 1272.38±144.07 grams (Males: 1322.47 ± 140.22 grams and Females: 1221.27 ± 129.55 grams). The normal weight of the brain is between 1300 to 1400 grams for Caucasian population.^7^ The weight of any human organ is found to be dependent on race, age, sex, environment, and socioeconomic conditions hence there are differences among organ weight in people residing in different countries.^8^ The normal reference range of brain weight in one population is not applicable to another population.^1,3,4,9^ To date no study in this regard has been done in Nepalese autopsy setting. It was thus necessary to derive mean brain weight for Nepalese deceased because incorrect reference values may result in misinterpretation of findings during autopsy.

Results from the present study were almost similar to findings in adult Africans, where the mean brain weight was 1280 grams.^9^ Studies done in the Chandigarh region of India also revealed similar values of mean brain weight in corresponding age groups.^2^ The reason for this similarity may be identical nutritional and environmental conditions between Nepal and India.

Contrasting to this, another study in the South African population revealed much higher brain weights (1404.82 ± 145.07 grams for males and 1267.13 ± 163.96 grams for females)^4^ that were in accordance to a study involving 699 deceased (Mean weight: 1318± 139.71 grams).^10^ Another study done in the Solapur region of India reveals slightly lower values of brain weight for both males and females by almost 100 grams^5^ which was consistent with other studies of a similar kind.^8,11,12^

Present study revealed maximum attainment of brain weight in the 21 to 30 years age group (1371.66±118.66) and gradual decline thereafter for both sexes. Similar observations were seen in NorthWest Indian population^2^ but with minimal differences in weight among different age groups, as opposed to the present study, where the difference is more.

One study had shown that loss of brain weight occurs ever earlier at the age of 20 years.^7^ Another study found that brain weight was increasing up to the mid-forties in both sexes.^1^ A study done on age-related changes to the structure of the brain demonstrated that a decline in mean brain weight is seen after 6^th^ decade.^13^ While another study found gender differences in age of attainment of maximum brain weight (30-39 years for male and 60-69 years for female).^5^ Hence there is wide variation in the findings of many studies regarding the age of attainment of maximum brain weight. Almost all the studies on brain weight reveal the mean male brain weight to be higher than that of female which is in accordance with this study.^3,6,8,10^

The limitation of this study was that it had a small sample size done in a single tertiary care centre. Selection of participants was not done randomly. So, the results might not be generalizable to wider populations.

## CONCLUSIONS

The weight of the brain derived from the present study is found to have variable values in comparison to other studies done in similar settings. The time of attainment of maximum brain weight is also highly variable compared to different studies. Other studies with a larger sample size consisting of autopsy cases from all over Nepal are recommended so that a normal reference range for organ weights can be established for Nepalese populations.
